# Needlestick injuries: a density-equalizing mapping and socioeconomic analysis of the global research

**DOI:** 10.1007/s00420-020-01547-0

**Published:** 2020-05-05

**Authors:** David A. Groneberg, Hannah Braumann, Stefan Rolle, David Quarcoo, Doris Klingelhöfer, Axel Fischer, Albert Nienhaus, Dörthe Brüggmann

**Affiliations:** 1 Institute of Occupational Medicine, Social Medicine and Environmental Medicine, Goethe-University, Frankfurt, Germany; 2 Institute of Occupational Medicine, Charité, Universitätsmedizin Berlin, Free University and Humboldt-University, Berlin, Germany; 3 Competence Centre for Epidemiology and Health Services Research for Healthcare Professionals (CVcare), University Medical Centre Hamburg-Eppendorf (UKE), Hamburg, Germany; 4 Department of Occupational Medicine, Hazardous Substances and Public Health (AGG), Institution for Statutory Accident Insurance and Prevention in the Health and Welfare Services (BGW), Hamburg, Germany; 5 Department of Obstetrics and Gynecology, Keck School of Medicine of USC, Los Angeles, CA USA

**Keywords:** Sharp injuries, Wounds, Density-equalizing mapping, Socioeconomic analysis, Bibliometrics

## Abstract

**Background:**

Needlestick injuries have caused a deleterious effect on the physical and mental health of millions of health-care workers over the past decades, being responsible for occupational infections with viruses such as HIV or hepatis C. Despite this heavy burden of disease, no concise studies have been published on the global research landscape so far.

**Methods:**

We used the New Quality and Quantity Indices in Science platform to analyze global NSI research (*n* = 2987 articles) over the past 115 years using the Web of Science and parameters such as global versus country-specific research activities, semi-qualitative issues, and socioeconomic figures.

**Results:**

Density-equalizing mapping showed that although a total of *n* = 106 countries participated in NSI research, large parts of Africa and South America were almost invisible regarding global participation in NSI research. Average citation rate (cr) analysis indicated a high rate for Switzerland (cr = 25.1), Italy (cr = 23.5), and Japan (cr = 19.2). Socioeconomic analysis revealed that the UK had the highest quotient *Q*_GDP_ of 0.13 NSI-specific publications per bill. US-$ gross domestic product (GDP), followed by South Africa (*Q*_GDP_ = 0.12). Temporal analysis of HIV versus hepatitis research indicated that NSI-HIV research culminated in the early 1990s, whereas NSI-hepatitis research increased over the observed period from the 1980s until the last decade.

**Conclusion:**

Albeit NSI research activity is generally increasing, the growth is asymmetrical from a global viewpoint. International strategies should be followed that put a focus on NSI in non-industrialized areas of the world.

**Electronic supplementary material:**

The online version of this article (10.1007/s00420-020-01547-0) contains supplementary material, which is available to authorized users.

## Background

As elegantly stated by recent reviews, the risks of needlestick injuries (NSI) may have fallen over the past three decades, but even in the presence of guidelines, they still occur frequently (King and Strony 2019). Health-care workers have the highest risk of experiencing needlestick injuries, but also other occupations are endangered (King and Strony 2019; Mona et al. 2019). When injured, the individual clinical, psychological and economic burden can be substantial (Cooke and Stephens 2017).

As previously stated, the rates of NSIs have been estimated to vary between 14.9 and 69.4% in health-care workers, depending on different settings, countries, and methodologies (Cooke and Stephens 2017). Hence, research in this area is crucial and knowledge about previous research is important to plan future strategies. Since there are no data existing on the international architecture of NSI research available, the present study used the NewQIS project platform (Groneberg-Kloft et al. 2009a) to construct the first picture of global NSI research strategies. We aimed to (1) perform an in-depth analysis of the global NSI research activities and to (2) visualize a variety of figures related to NSI research by the use of density-equalizing procedures proposed by Gastner and Newman (Gastner and Newman 2004).

## Methods

### Methodical platform

To analyze NSI research, we used the New Quality and Quantity Indices in Science (NewQIS) platform termed NewQIS (Groneberg-Kloft et al. 2009a, b), ranging from public and global health issues (Groneberg et al. 2016; Trost et al. 2018) to fields such as internal medicine (Schoffel et al. 2018), surgery (Schoffel et al. 2016, 2017) or gynecology and obstetrics (Bruggmann et al. 2016a, b). Since its foundation, about 80 peer-reviewed studies have been published using NewQIS. As data source, we used the Web of Science (Clarivate) since this database enabled us to calculate various citation parameters (Gerber et al. 2014).

### Search algorithm

**Fig. 1:**
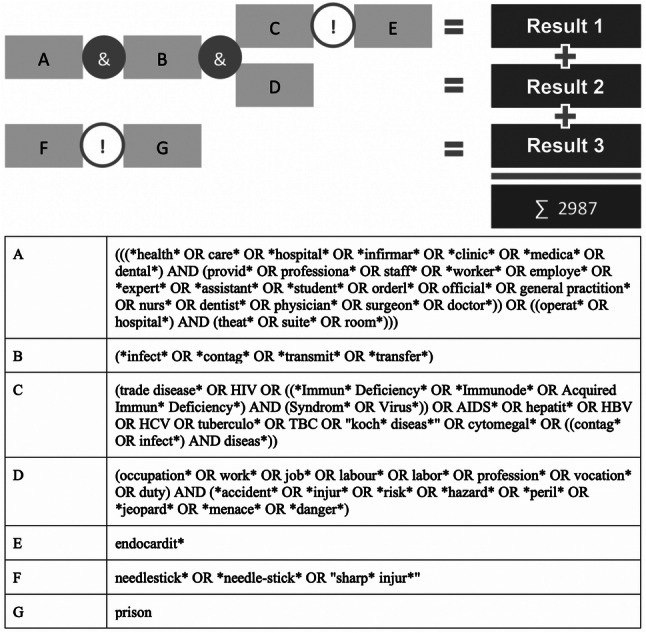
Schematic illustration of the search term

To cover studies related to occupational needlestick injuries, we constructed a detailed search algorithm, illustrated in Fig. 1.

Using this term, we performed a topic search in the period 1900–2014. As in earlier studies, the period after 12–31-2014 was excluded to avoid incomplete data acquisition.

### Data analysis and categorization

As earlier stated, NewQIS enables to analyze and visualize a wide range of different parameters (i.e., the time of publication, the originating countries, the attributed subject categories, the citations). With these raw data, further parameters such as country-specific h-indices (HI) of the present set of publications were calculated. These HI are not related to single authors, but to the countries. Also, country-specific citation rates of NSI-related research of each country were calculated (CR, total country-specific citations numbers per total numbers of country-specific NSI publications) (Hirsch 2005). In a next step, the numbers of collaborating countries in collaborating articles were also analyzed.

### Socioeconomic and epidemiologic analysis

Absolute research activity is important to assess the overall contribution of a single country toward the global research efforts in the areas of NSI. However, there are vast socioeconomic differences between single countries. Therefore, the research activity data should also be related to socioeconomic figures of each country to be able to interpret the relative contributions. We approached this issue by relating NSI research activity to the (1) total economic power index “gross domestic product” (GDP) per billion US-$, (2) GDP per capita, and (3) country population sizes (*World Factbook* (World Economic Outlook Database 2013)).

### Density-equalizing map projections of NSI research

We here used the approach of density-equalizing map projections (DEMP) established by Gastner and Newman in 2004 (Gastner and Newman 2004). To construct the DEMPs, we introduced several NSI-specific parameters to the algorithm including total numbers of NSI-related publications, country-specific citation rates of NSI-related research and others.

### International NSI networks of research

As stated earlier, NewQIS also assesses global collaborative networks. Using a methodological approach which was previously described, all affiliations of NSI-related articles were analyzed for their originating countries. Then, chart diagrams were generated to visualize the network (Mund et al. 2014).

### Sub-analysis of NSI-acquired diseases

In two separate analyses, we assessed the research activities for the most prominent NSI-related acquired diseases—HIV and hepatitis. In specific, the search term listed in Fig. 1 was separated into two different, HIV- and hepatitis-specific terms as listed in online supplement 1. The metadata was analyzed for chronologic development and countries of research origin.

## Results

### General parameters

**Fig. 2:**
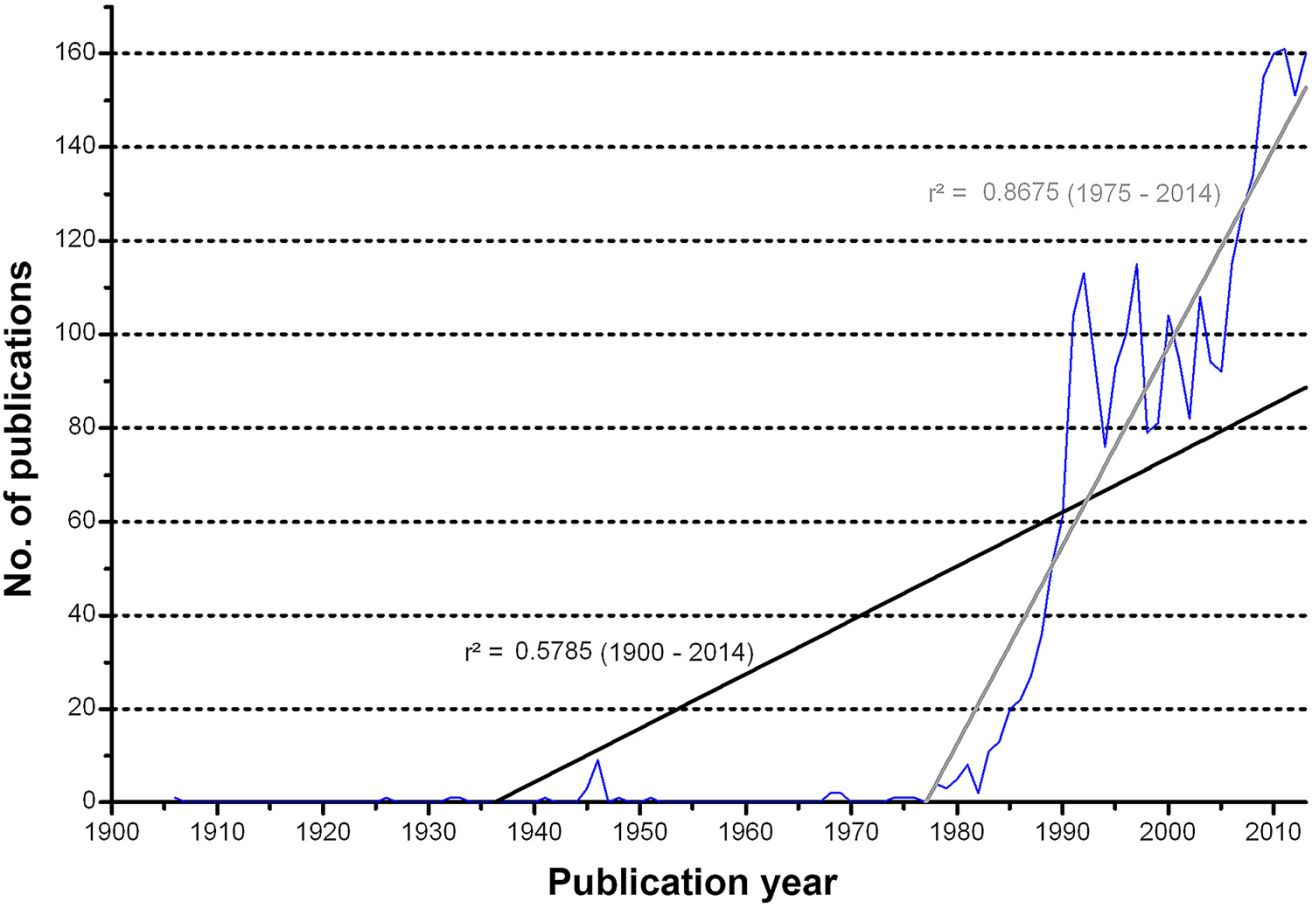
Development of the number of publications with linear regression at two times, *p* < 0.000*** (Spearman)

The first publication on NSI was identified for the year 1906, but until 1982, only a moderate activity was present with less than 20 articles being published annually. In the 1980s, an increase was present and beginning of the 1990s, more than 100 annual articles on NSI were published for the first time. In total, 2987 articles were identified. More than 99% of all articles were published after the year 1975. A regression analysis of the investigated research interest over time reveals a high coefficient of determination (*r*^2^ = 0.87) since 1975 (Fig. 2).

### NSI country-specific analysis

**Fig. 3:**
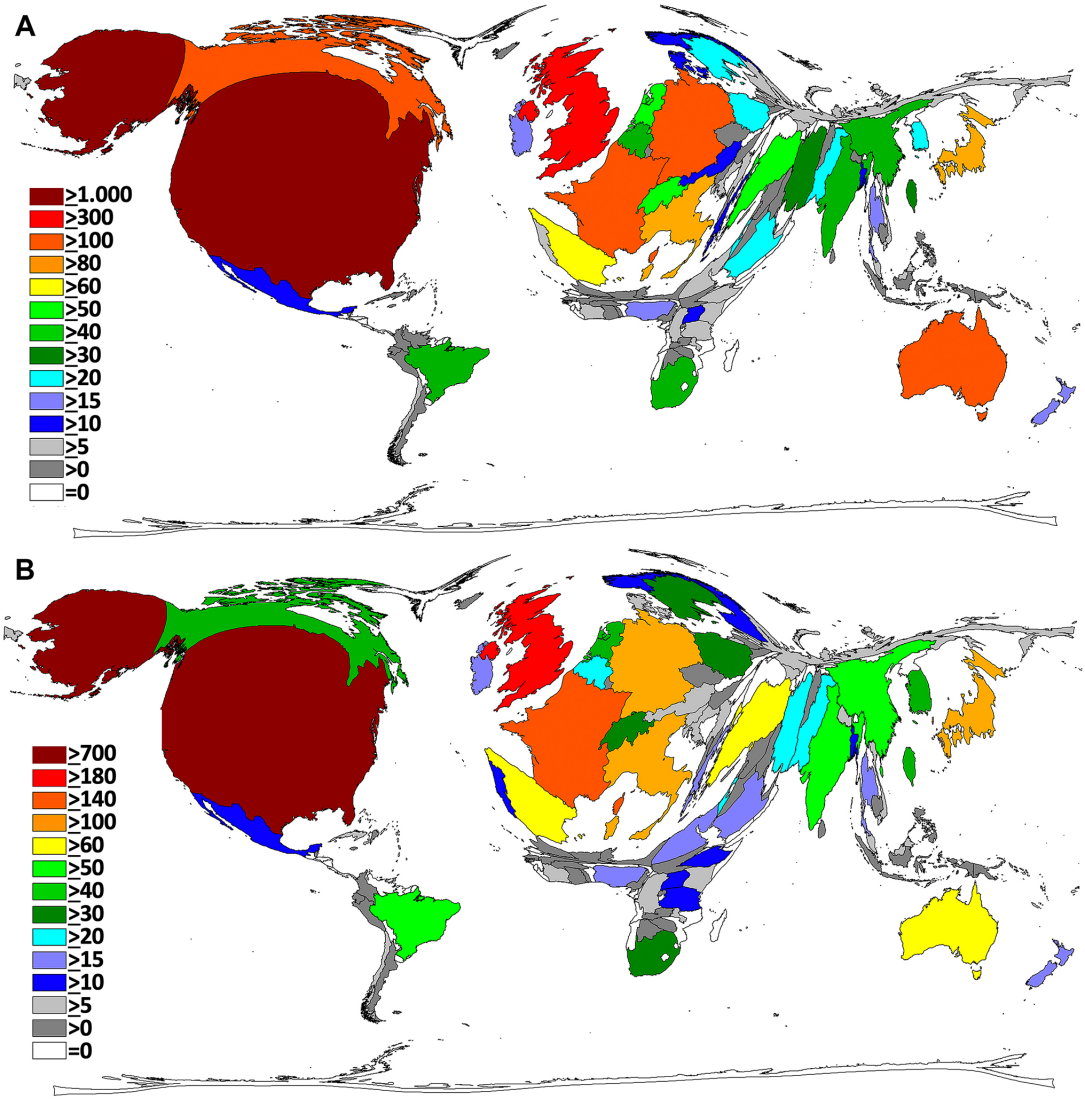
Country’s performance. **a** Number of publications. **b** Number of institutions

In total, 106 countries published NSI-related articles. The nation with the highest level of NSI research activity was the USA with *n* = 1,237 articles. Next, Great Britain showed an activity of 317 NSI-related articles, followed by Canada (*n* = 138), Australia (*n* = 132), Germany (*n* = 131) and France (*n* = 113). When all data are transferred to a DEMP algorithm, a world map distorted toward North America and Western Europe is present. In the Eastern Hemisphere, Japan and Australia are prominent. Australia is the only country south of the equator with an activity over 100 NSI articles—indicating a strong north–south slope (Fig. 2). A similar landscape is present, when the number of NSI publishing institutions/affiliations per country is analyzed (Fig. 2): The USA is the leading country with *i* = 739 different affiliations, followed by Great Britain (*i* = 183), France (*i* = 148), Italy (*i* = 129), Germany (*i* = 110), and Japan (*i* = 109) (Fig. 3).

### NSI citation analysis

**Fig. 4:**
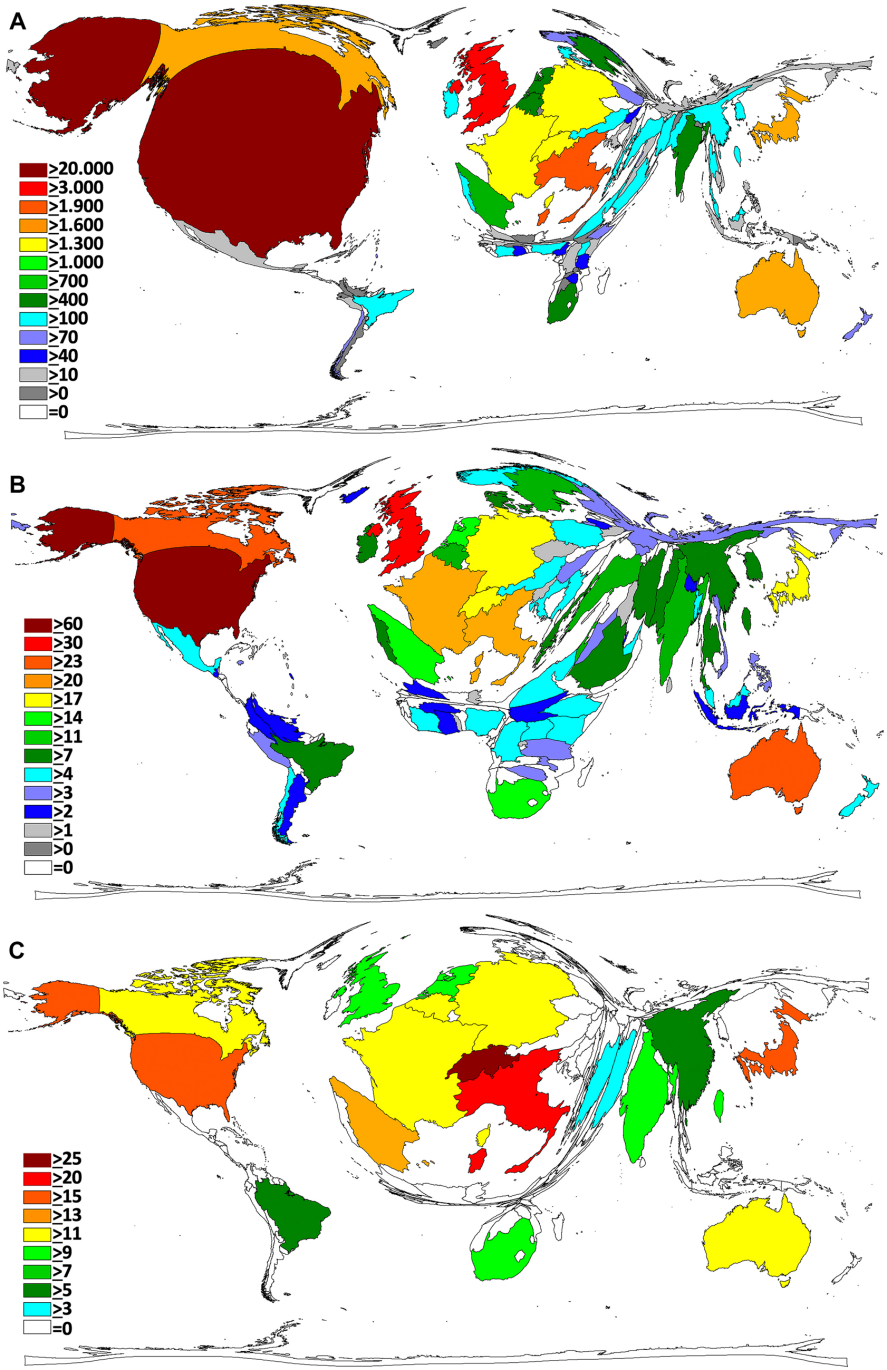
Citation parameters. **a** Number of citations. **b** Modified h-Index. **c** Citation rate (threshold = 30 publications)

The analysis of the crude parameter of the total citation count followed the picture of the total publication activities with only small variations being present: The USA was at the lead position with *c* = 23,164 citations. It was followed by Great Britain (*c* = 3208 citations), Italy (*c* = 1977), Canada (*c* = 1681), Japan (*c* = 1669), Australia (*c* = 1618), and Germany (*c* = 1545) (Fig. 4a). As a second citation readout, the country-specific Hirsch indices (coHI) were assessed for the presently identified NSI-related publications. In this assessment, DEMP calculations led to a global architecture similar to the total citation count projections with the USA at the lead position with a coHI of 68 publications being at least cited 68 times. The USA was followed by the UK (coHI = 30), Canada (coHI = 24), Australia (coHI = 23), France and Italy (both a coHI of 22), Germany, Japan and Switzerland (all a coHI of 19) and the Netherlands (coHI = 15). The only African country with a coHI more than 10 is South Africa (coHI = 14) (Fig. 4b).

A complete different global architecture was found when the NSI publications were analyzed for the average citation rates with a threshold of at least 30 publications: Here, Switzerland was ranked first (cr = 25.1), followed by Italy (cr = 23.5), Japan (cr = 19.2), USA (cr = 18.7), and Spain (cr = 13.3) (Fig. 4c).

### Socioeconomic analysis of NSI research activities

Countries with a high level of economic prosperity are able to allocate more funding toward NSI research from a financial point of view. Therefore, we also assessed NSI research in relation to socioeconomic figures. We first assessed the correlation between the country-specific number of NSI-related articles and the countries’ gross domestic product in bill. US-$ (GDP, Table 1). When applied for all countries with at least 20 specific articles related to NSI, the UK is ranked highest with a quotient Q_GDP_ of 0.13 NSI-specific publications per bill. US-$ GDP. The UK is followed by South Africa with a quotient of Q_GDP_ = 0.12, and Iran (Q_GDP_ = 0.11). Next, Pakistan (Q_GDP_ = 0.1), Switzerland (Q_GDP_ = 0.09), Australia, Belgium, Canada, and Israel follow with a Q_GDP_ of 0.08. The USA, Turkey and the Netherlands have a Q_GDP_ of 0.07), followed by Sweden and Poland (Q_GDP_ = 0.05), and Spain (Q_BIP_ = 0.04).

**Table 1:** Ranking of publication numbers (*n*) per gross domestic product in bill

Country	*n*	GDP	*n*/GDP	Expenditures for R&D in % GDP
UK	317	2521	0.13	1.6
South Africa	43	351	0.12	n/a
Iran	39	369	0.11	n/a
Pakistan	23	237	0.1	0.3
Switzerland	57	650	0.09	3.0
Australia	132	1561	0.08	2.3
Belgium	40	508	0.08	2.3
Canada	138	1827	0.08	1.6
USA	1237	16,800	0.07	2.8
Turkey	58	820	0.07	0.9
Netherlands	55	800	0.07	2.0

US dollar (GDP) (World Economic Outlook Database 2013), threshold = 20 publications, Research & Development (R&D) 2013 (StatistischesBundesamt 2016), *n/a* not accessible

In a next step, we assessed the correlation between the country-specific number of NSI-related articles and the countries’ gross domestic product per capita (Table 2): In this ranking with a threshold of at least 20 specific articles, India is ranked first with 0.033 NSI-specific articles per GDP/capita, followed by the USA and Pakistan.

**Table 2:** Country’s ranking of the number of publications (*n*) per gross domestic product per capita (GDP per capita) (World Economic Outlook Database 2013) in bill

Country	*n*	GDP per capita	*n*/GDP per capita
India	49	1499	0.033
USA	1237	53,143	0.023
Pakistan	23	1299	0.018
Iran	39	4763	0.008
UK	317	39,337	0.008
South Africa	43	6618	0.006
China	42	6807	0.006
Turkey	58	10,946	0.005
Brazil	47	11,208	0.004
Germany	131	45,085	0.003
France	113	41,421	0.003

US dollar, threshold = 20 publications

In a final assessment, NSI research was related to population sizes. Here, Switzerland was ranked first with a calculated number of 7.05 NSI-related articles per million of inhabitants, followed by Australia, the UK, Canada and the USA (Table 3).

**Table 3:** Country’s ranking of the number of publications (*n*) per mill. inhabitants, threshold = 20 publications

Country	Inhabitants	*n*/Inhabitants in mill
Switzerland	8,081,482	7.05
Australia	23,130,900	5.71
GB	64,097,085	4.95
Canada	35,158,304	3.91
USA	316,128,839	3.91
Belgium	11,195,138	3.57
Netherlands	16,804,224	3.27
Sweden	9,592,552	2.92
Israel	8,059,400	2.73
France	66,028,467	1.71

### Network analysis of international NSI research activities

**Fig. 5:**
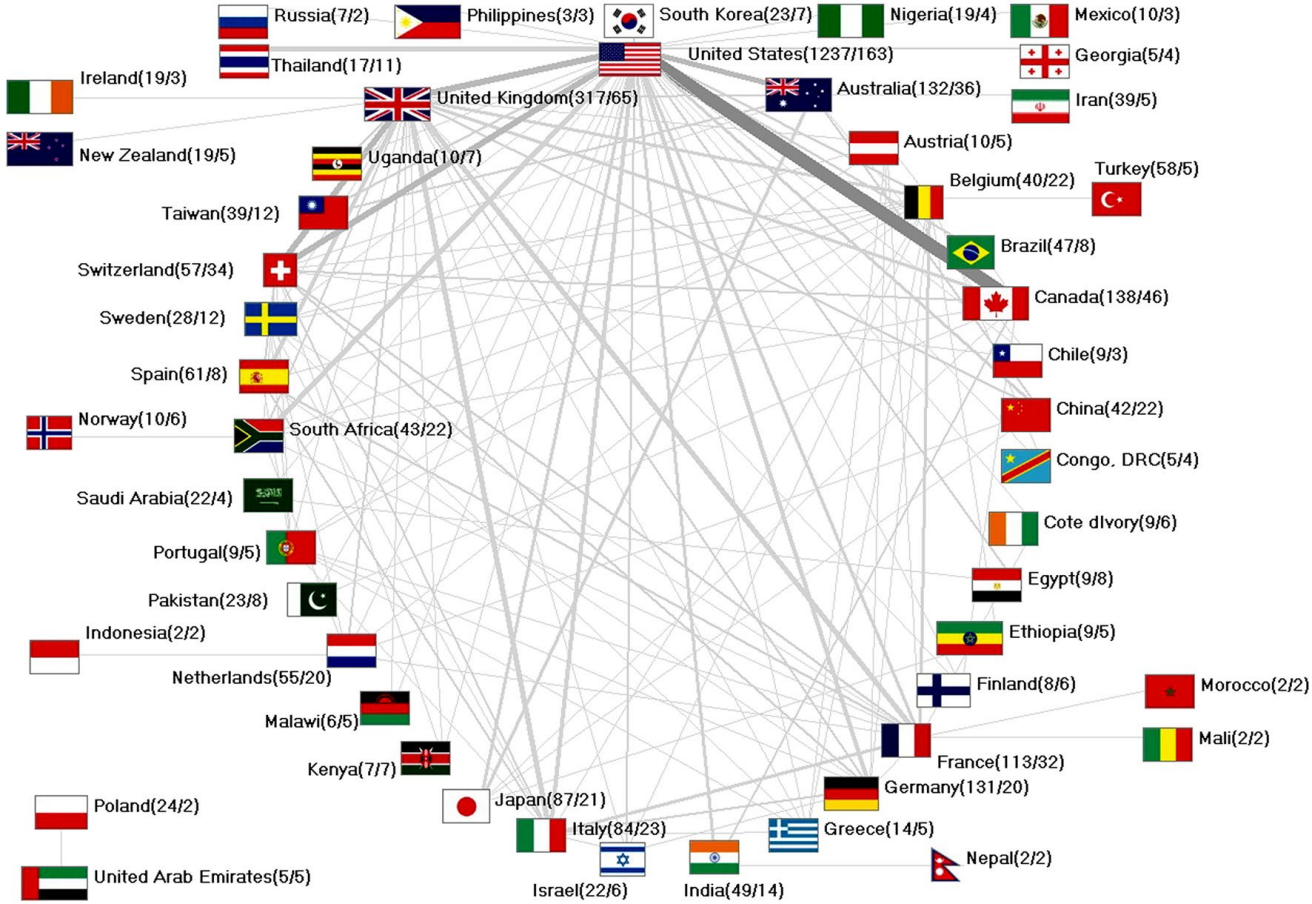
International collaborations, number in brackets (no. of publications/no. of collaboration articles)

In total, there were 335 collaborative articles (*n*_coop_) present. About 80% were bilateral cooperations (*n*_coop_ = 268). Trilateral publications were present in with *n*_coop_ = 45 cases, followed by collaborations of four countries (*n*_coop_ = 13 and five countries (*n*_coop_ = 4). There were 1,325 US collaboration articles, followed by UK (*n*_coop_ = 360), French (*n*_coop_ = 331), Chinese (*n*_coop_ = 324), German (*n*_coop_ = 319), and Canadian (*n*_coop_ = 264) collaborations (Fig. 5).

The leading nation was the USA with a total of 163 collaboration publications. The most prominent collaboration was between US-American and Canadian scientists (Fig. 4), followed by US–Swiss and US–UK collaborations.

### NSI research area analysis

**Fig. 6:**
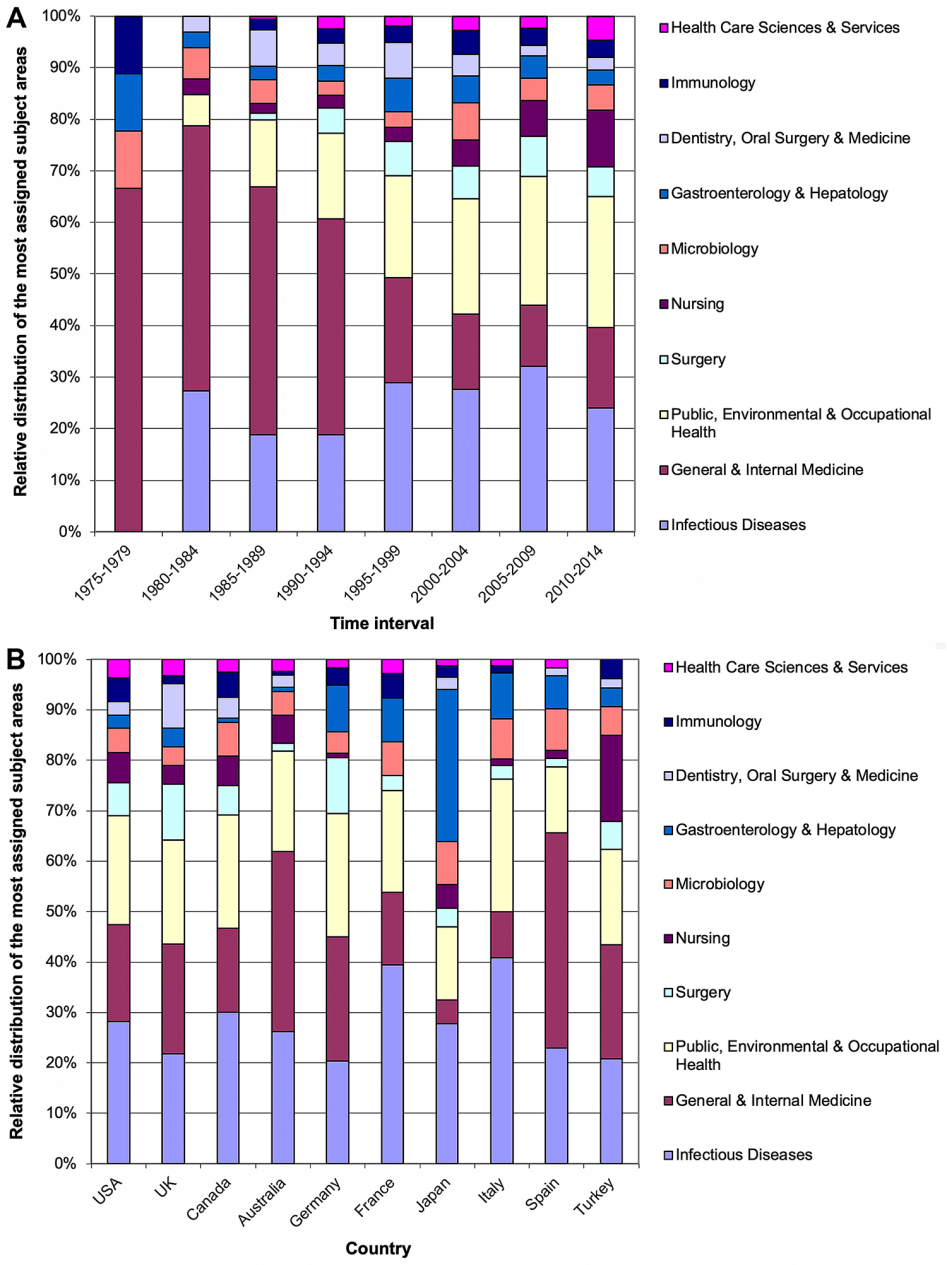
Subject areas. **a** Relative distribution of the subject areas in 5-year intervals from 1975 to 2014. **b** Relative distribution of the most assigned subject areas in the most publishing countries

Subject category analysis of published NSI research showed an increase in *Public, Environmental and Occupational Health-* topics related to NSI over the past decades (Fig. 6a). Also, the field of nursing gained research activity in the period of 2010–2014. When single countries were analyzed for differences in their research focuses, a heterogeneous picture was present (Fig. 6b). The most apparent difference was found for Japan with an emphasis on the field of gastroenterology and hepatology. Besides, Turkey was the country with the highest proportion of NSI research related to the field of nursing.

### Sub-analysis of global research activities on NSI-acquired diseases

**Fig. 7:**
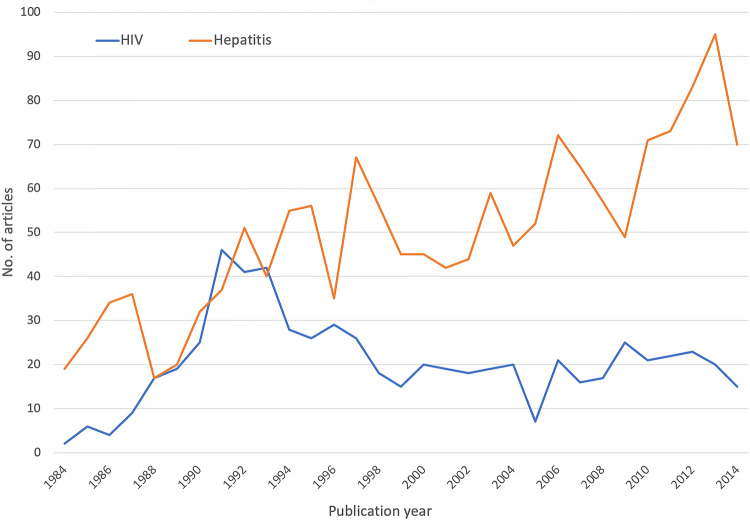
Temporal sub-analysis of NSI-acquired diseases (HIV and hepatitis)

A temporal analysis and comparison of research activities focused on NSI and HIV or NSI and hepatitis shows that there are major differences present. Whereas NSI research, specifically on HIV, culminated in the beginning of the 1990s, NSI-research focused on hepatitis increased over the observed period from the 1980s until the last decade (Fig. 7). When a sub-analysis of countries’ research activities was performed (Table 4), the USA dominated both NSI research on both acquired diseases, followed by the UK. It is noteworthy that South Africa also appeared in the top ten most active countries concerning NSI and HIV. By contrast, the only Asian country which is present in the overall activity ranking, Japan, does not belong to the most active NSI-HIV-focusing countries. However, it is present in the NSI-hepatitis top ten ranking countries.

**Table 4:** Sub-analysis of countries’ research activities on NSI-acquired diseases

Country ranking	Total *n*	Country ranking	HIV	Country ranking	Hepatitis
USA	1237	USA	324	USA	490
UK	317	UK	68	UK	185
Canada	138	France	32	Germany	89
Australia	132	Canada	30	France	87
Germany	131	Germany	24	Italy	80
France	113	South Africa	24	Australia	67
Japan	87	Australia	20	Canada	60
Italy	84	Netherlands	18	Japan	51
Spain	61	Spain	14	Spain	49
Turkey	58	Belgium	13	Switzerland	36

Left column: total number of articles. Middle column: number of articles related to NSI and HIV. Right column: number of articles related to NSI and hepatitis

## Discussion

NSIs are omnipresent around the globe and endanger the health and life of hundreds of thousands of health-care workers. As stated before, they generate a large amount of indirect, direct, potential, and intangible costs, which possibly increase over time (Mannocci et al. 2016). Despite this enormous burden of disease, no concise visualization of the global research efforts has been established so far. Why? Probably, because NSIs are not just a single entity such as a disease or a pathogen, but an accident, resulting from a series of failures, which can be analyzed from different perspectives: determinants, characteristics, distribution, consequences, prevention of accidents, and prevention of consequences. Therefore, the present results need to be interpreted with caution. Our approach aimed to visualize global research activities in the field of NSI and related areas of science. To approach this aim, metadata were used from the Web of Science between 1900 and 2014 and 2,987 publications were identified which were specifically related to NSI. Interestingly, almost all NSI-related articles (99.2%) were published between the years 1975 and 2014, indicating only a very minimal activity in the decades before. The reason for this increase after 1975 is based upon two findings: observations that (a) hepatitis B (first articles appearing in 1975–80), and then (b) HIV (first documented case in literature in 1984) could be acquired through a needlestick. One might argue that due to the minimal activities more than three decades ago, the analysis should have been limited to, i.e., the past decades. However, including a time span from 1900 to 1975 directly illustrates the lack of global NSI activities. This information might be useful for a placement on the historical continuum of medical sciences.

The USA plays a dominant role in country-specific analyses with the highest number of articles published, the largest number of institutions of higher education as well as the highest modified h-Index. Furthermore, the USA possesses the most national and international collaborations among all nations. How can these data be interpreted? First of all, it is important to discuss these results with regard to the overall global research performance and general trends. To achieve this, the presently observed increase in NSI research activities needs to be related to the increase in global research activities, in specific, biomedical research activities. It is wrong to take the growth of databases such as the presently used Web of Science as a precise tool for increase in research activities (since no existing database captures everything) (Noorden 2014). However, it may serve as a proxy (Bornmann and Mutz 2014). By analyzing more than 755 million cited references in 38 million publications from 1980 to 2012, Bornmann and Mutz estimated the growth of research for 2012 between 8 to 9% (Bornmann and Mutz 2014). Although only restricted to WoS data, our analysis shows for NSI a growth rate which is not constantly increasing, but varying between years, especially in the 1990s to the 2000s. This is an uncommon picture, because usually the growth is more or less linear, as previously demonstrated (Noorden 2014; Bornmann and Mutz 2014).

How is the global architecture of NSI research? As stated above, the USA is by far dominating the international efforts in NSI research. Is this a common picture—yes, there is a multitude of other bibliometric studies that demonstrated a unique leading role of the USA in almost every area of science.

But how is the pattern of leading countries apart from the USA? Does this ranking follow any previously known pattern? Our ranking with the UK at position 2, followed by Canada (*n* = 138), Australia (*n* = 132), Germany (*n* = 131) and France (*n* = 113) shows a global architecture that is similar to those of many other fields of biomedicine. But is this pattern specific for research conducted in the area of viral diseases?

There are a number of studies that addressed global research activities on infectious diseases so far: both NSI-relevant infectious diseases/agents such as HIV or hepatitis virus and other diseases such as Ebola or influenza. In general, there are two global patterns present: a pattern for infectious diseases with a large burden of disease in industrialized countries and/or infectious diseases with the possibility of vaccination such as hepatitis (Schmidt et al. 2013), HPV (Bruggmann et al. 2018), or influenza (Fricke et al. 2013) and a pattern for diseases that are more common in non-industrialized or tropical countries such as leishmaniasis (Al-Mutawakel et al. 2010). The present NSI research activity pattern is more or less similar to the ones reported for HIV or hepatitis (Schmidt et al. 2013). It differs from the patterns of diseases linked to viruses such as yellow fever (Bundschuh et al. 2013) and MERS-CoV (Zyoud 2016). Here, countries such as Brazil and India appear in the top ten ranking most active countries.

Next to these continental differences, one might also focus future studies upon further variations, i.e., between countries of the European Union, which enjoy shared health and safety legislation, and other regions of the Northern globe. In this respect, it has been recently demonstrated with regard to psychosocial risks and violence in the workplace that there are important differences between these regions (Chirico et al. 2019). A similar study set up using databases such as LEGOSH” of the International Labour Office (ILO) might add important new insights into the understanding of global and regional NSI research differences.

We also related overall NSI research figures to socioeconomic features including GDP or population sizes. A limitation of this approach is the constant change in these socioeconomic parameters. However, they are commonly used as proxies to relate research productiveness to socioeconomic features (Chong et al. 2016; Wang and Zhao 2018).

Apart from the country-specific analysis of research activities, it is also of major importance to analyze the subject areas in which NSI research is performed and published. In contrast to global research activities, e.g., on hepatitis (Schmidt et al. 2013), or HPV (Bruggmann et al. 2018), we here found for NSI that the field of *Public, Environmental and Occupational Health* gained importance in the past decades. In the final 5-year period of analysis, this field was among the most active areas of NSI research. This points to the importance of NSI for this area of biomedicine.

Obviously, NSI cases among health-care workers have also occurred in countries where the HIV epidemic was widespread (i.e., African countries), but they were less likely to be identified due to the ongoing epidemic in the general population, and the lack of specific surveillance at the beginning of the epidemic. In our sub-analysis, South Africa was detected to belong to the top ten most active countries concerning HIV-specific NSI research. Other countries, such as Japan, with a significant prevalence of HCV in the general population, observed cases of hepatitis C linked to NSIs after the identification of a specific antibody test to detect seroconversion in the early 1990s, and this is why papers appear in gastroenterology and hepatology journals. This is visualized by our NSI research area analysis in which Japan is very prominent in the field of gastroenterology and hepatology. Also, in our sub-analysis, Japan appeared in the top ten activity ranking of specific NSI-hepatitis research.

The different waves of publications are in relationship with the different subjects related to NSIs (and countries) as demonstrated here. The evolution of these areas with regard to NSI occurred over time, i.e., pathogens acquired, effects of the first preventive measures (universal precautions, banning of recapping, sharps containers, hepatitis B vaccination), post-exposure prophylaxis against HIV, introduction of safety devices, and impact of laws and regulations supporting preventive measures.

## Conclusions

We here present data on activity of NSI research in the twentieth and twenty-first centuries. Our data clearly show that there is a heavy global asymmetry with the Southern globe being underrepresented to a major extent. Although the area of public and occupational health—being underpresented concerning research activity in the majority of other medical areas—is omnipresent in NSI research. This study points to the need to foster NSI research activities in non-industrialized countries in which health-care personnel are crucial for public health.

## Electronic supplementary material

Below is the link to the electronic supplementary material.

Supplementary file1 (DOCX 615 kb)

## Data Availability

The bibliometric data are owned by and have been obtained from the Web of Science database. Any researcher with access to the Web of Science database can obtain the data using the methods described in the paper. Readers who do not have access to Web of Science should contact Clarivate Analytics to obtain a license. As per Clarivate Analytics terms of use, the authors may also be able to provide limited access to data, subject to their agreement.
