# Supplementary material for: Needlestick injuries: a density-equalizing mapping and socioeconomic analysis of the global research
Source: Int Arch Occup Environ Health. 2020 May 5;93(8):995–1006. doi: 10.1007/s00420-020-01547-0 (PMC7199875; doi:10.1007/s00420-020-01547-0)

**Needle stick injuries: A density equalizing mapping and socioeconomic analysis of the global research**

Short title: needle stick density equalizing mapping

Online Supplement:

Sub-analysis on NSI and HIV.


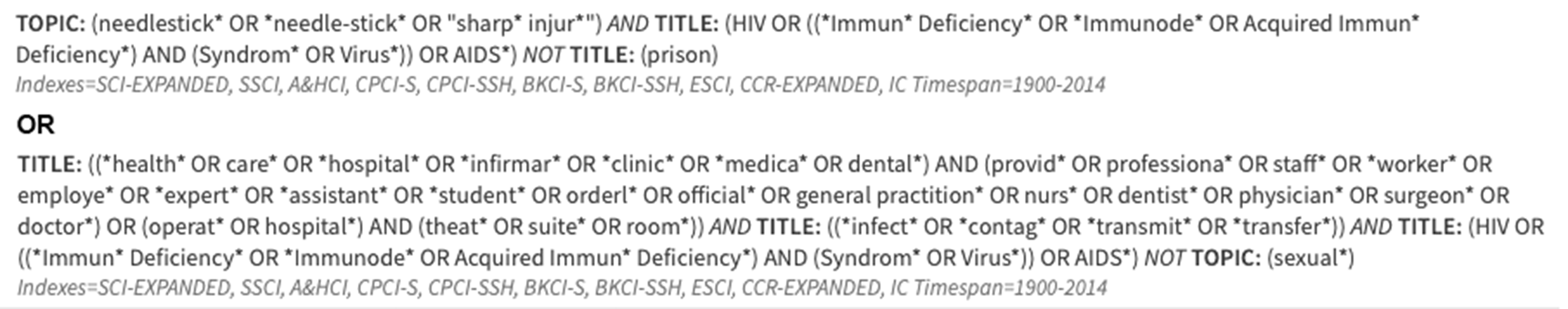


Sub-analysis on NSI and hepatitis.


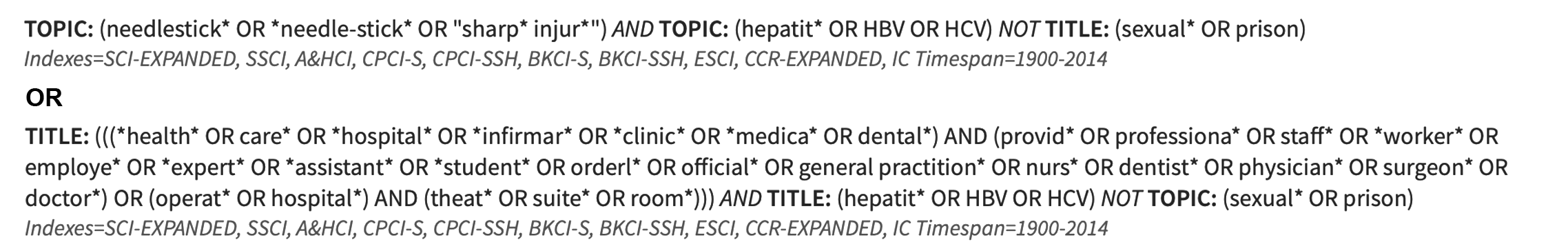

Supplement: Supplementary file 1 — Supplementary file1 (DOCX 615 kb) [file 420_2020_1547_MOESM1_ESM.docx]
